# Acceptability of risk-based breast cancer screening among professionals and healthcare providers from 6 countries contributing to the MyPeBS study

**DOI:** 10.1186/s12885-025-13848-z

**Published:** 2025-03-15

**Authors:** Alexandra Roux, Lucile Hervouet, Francesca Di Stefano, David P. French, Livia Giordano, David Ritchie, Marie-Eve Rougé Bugat, Debbie Keatley, Rachel Cholerton, Lorna McWilliams, Paolo Giorgi Rossi, Corinne Balleyguier, Michal Guindy, Fiona J. Gilbert, Jean-Benoit Burrion, Marta Roman, Cécile Vissac-Sabatier, Daniel Couch, Suzette Delaloge, Sandrine de Montgolfier, On behalf of the MyPeBS Investigators and the MyPeBS Consortium

**Affiliations:** 1 Aix Marseille Univ, Inserm, IRD, ISSPAM, SESSTIM, Marseille, France; 2 IRIS Institut de recherche interdisciplinaire sur les enjeux sociaux (UMR 8156 CNRS - 997 INSERM - EHESS - UPSN), Campus Condorcet, Aubervilliers, France; 3 Piedmont Center for Cancer Epidemiology and Prevention, CPO, AOU Città della Salute e della Scienza di Torino, Turin, Italy; 4 Manchester Centre for Health Psychology, University of Manchester, Manchester, UK; 5 European Cancer Leagues, Brussels, Belgium; 6 Département Universitaire de Médecine Générale, Université Toulouse 3 Paul Sabatier, Toulouse, France; 7 Independent Cancer Patient’s Voice, London, UK; 8 AUSL - IRCCS di Reggio Emilia, Reggio Emilia, Italy; 9 Institut Gustave Roussy, Villejuif, France; 10 Assuta Medical Centers, Tel Aviv, Israel and Ben Gurion University, Beersheba, Israel; 11 University of Cambridge, Cambridge, UK; 12 Institut Jules Bordet, Bruxelles, Belgium; 13 IMIM (Hospital del Mar Research Institute), Barcelona, Spain; 14 Unicancer, Paris, France; 15 University of Paris Est Créteil, Créteil, France

**Keywords:** Risk-based screening, Professionals, Healthcare providers, Breast cancer screening, Training, MyPeBS trial, Acceptability

## Abstract

**Background:**

To evaluate the acceptability of a risk-based breast cancer screening (BCS) strategy among professionals involved in MyPeBS study in 6 countries.

**Methods:**

After qualitative interviews, a questionnaire was built with a Delphi method: to evaluate professionals’ basic understanding, satisfaction and reactions to each stage of the trial, opinions on BCS and its future. The questionnaire was distributed by emailing 698 investigators, who forwarded it to all categories of professionals involved in trial recruitment (physicians, medical secretaries, nurses, and mammography technicians). Descriptive statistics were used to summarize views on acceptability.

**Results:**

Among the 198 respondents, most declared being at ease with the trial design and the concept of breast cancer risk estimation. They were mostly comfortable explaining the different trial steps, communicating risk estimation, and answering women’s questions. Some professionals were not comfortable explaining high (7.1%) and low-risk categories (9%) and did not feel sufficiently trained (26.5%). Although professionals were mostly confident about risk-based approaches and the potential of this to improve breast cancer screening (93.5%), 58% called for further validation of the risk-models to predict risk before implementation in population-based programs. They expressed concerns about the complexity of this screening strategy, stressing the need to properly inform the public and to train professionals in delivering risk assessment.

**Conclusion:**

This first study assessing the perspectives of professionals delivering risk-based BCS. As professional acceptability is key for successful implementation, training for all professionals and tools to help them communicate risk to women will be necessary to develop risk assessment in BCS.

**Trial registration:**

Study sponsor: Unicancer. My personalised breast screening (MyPeBS). Clinicaltrials.gov (2018) available at https://www.clinicaltrials.gov/ct2/show/NCT03672331.

**Supplementary Information:**

The online version contains supplementary material available at 10.1186/s12885-025-13848-z.

## Background

Breast cancer is the leading cancer cause of death among women worldwide [28]. Currently, breast cancer screening uses age as the primary criterion for eligibility (over 50 years) and is perform every two years in most European countries. Debates exist on the optimal screening interval, the benefits and harms of regular screening (false-positives, false-negatives, overdiagnosis, overtreatment, anxiety, difficulty to reach some persons concerned and unnecessary costs) [19, 22].

Since the risk of developing breast cancer varies widely among women (according to age, genetics, family and reproductive history, history of benign breast disease, BMI, hormones and other exposures), risk-based approaches have recently emerged as a promising strategy [17, 24, 26]. They require applying risk assessment to the whole screening-age population, stratifying the population into risk groups, and tailoring prevention and early detection interventions to each group according to risk [6, 12]. Despite expected benefits, clarification is needed on the ethical, legal and social implications (ELSI) of a risk-based screening program [7, 23], particularly regarding the acceptability of such risk-based strategy among women but also healthcare professionals.

Internationally, there are two ongoing studies analyzing the outcomes of risk-stratified screening in comparison to routine breast cancer screening: WISDOM and MyPeBS^1^(“My Personal Breast Screening”) [8]. MyPeBS is a randomized controlled trial currently running in six countries (Italy, France, Israel, the UK, Belgium, and Spain). For women randomized in the risk-based arm, screening frequency and methods depend on their individual 5-year predicted risk of invasive breast cancer (on health family history, mammographic density, age, lifestyle indicators, and genotyping including a validated polygenic risk score on DNA extracted from a saliva sample with 313 relevant polymorphisms) [27].

Previous literature shows that healthcare professionals (HCPs) have expressed doubts about the precision of statistical models of risk-prediction and the possibility of translating them into clinical risk categories. HCPs called for more evidence-based proof to implement it at a population level without causing any harm [2, 3, 20, 21, 30]. They questioned the feasibility and the constraints on national healthcare systems [2–4, 13, 15, 20, 29, 30]. HCPs were particularly concerned by delivering a low risk or high risk estimate. They also expressed doubts about the accuracy of the information provided by women with consequences on miscalculations of risk (Puzhko et al., 2019).

The present work evaluates the acceptability of the risk-based breast cancer screening strategy among a large diversity of professionals involved in the MyPeBS study in 6 countries. It should contribute to assess the chances of success of a potential future implementation of cancer risk assessment at a population level if risk-stratified screening was demonstrated to be preferable to current organized screening programs.

## Methods and data collection

### Preparatory work and Delphi procedure

Delphi methodology, to obtain consensus among a group of experts, was used to build this study’s questionnaire [11, 14]. Preparatory work to elaborate and validate the questionnaire was carried out between 2019 and 2021 with in-depth qualitative interviews (*n* = 15) by LH with professionals involved in the trial in France and short online interviews (*n* = 3) and question forms (*n* = 4) by AR with the principal investigators from each country of MyPeBS, so as, to assess differences in how the trial was conducted in the various centers and countries and to produce questions relevant to all professionals. The first version of the questionnaire was designed by the two main researchers of this sub-study (AR and SdM) and was submitted to the critical feedback of a Delphi team composed of six researchers involved in MyPeBS (FDS, DF, LG, LH, DR, MERB) and then reviewed by MyPeBS officials (CB and SD).

Delphi procedure was adapted with two rounds^2^ [1, 5, 10, 11, 14, 16]. The first version of the questionnaire was evaluated online in June 2021 by a group of experts (*n* = 14), representative of each country of the trial, and selected by each country’s primary investigator for their relevance to our study and their knowledge of the national implementation of the MyPeBS trial. For each question, members of the Delphi team had to indicate: if it seemed relevant to include the question in the final questionnaire (scale 0–9); if the wording was acceptable (scale 0–9); and to suggest other formulations (by open question). Only the questions that achieved a level of satisfaction among evaluators superior to 7 (out of 9) were chosen for the final questionnaire. A new version of the questionnaire was then elaborated and reviewed by the Delphi team (July 2021). The final English version was translated into French, Hebrew, Italian, Spanish, and Dutch, and subject to a second round of evaluation to check the accuracy of the translation (August - September 2021).

### Participants

The originality of the present approach is to collect data on all professionals involved in the trial regardless of their official status towards the trial. That includes study investigators officially in charge of recruiting women in the study, but also other professionals in contact with participating women and thus likely to provide them with information on the trial, whatever their level of implication or their place in the health/screening center’s hierarchy: physicians, medical secretaries, nurses, mammography technicians, local study coordinators or administrators, data or project managers, epidemiologists. The term “professionals” was used to encompass this diversity of professions involved in the trial, which is not limited to MyPeBS investigators. The main difference is that investigators had to follow a compulsory e-learning training to be allowed to recruit women for the trial^3^. Other professionals may have followed the e-learning training offered by MyPeBS, may have had other sources of training, or not training at all. The questionnaire was set up online (with Sphinx^®^ software) in 6 languages and then distributed to all declared study investigators by email (698 in November 2021 with 444 in France, 122 in Italy, 56 in Belgium, 47 in Israel, 19 in UK and 10 in Spain). Investigators were invited to transfer the invitation to all professionals of their staff involved in the trial. Participants were informed about GDPR compliance of data treatment and protection on the first page of the questionnaire. They had to informally consent to proceed further. Three waves of invitation were done (September 2021, *n* = 103 answers; November 2021, *n* = 75 answers; April 2022, to collect answers from centers that had recently begun recruiting women, *n* = 20). Given the diversity of national screening organizations in each country, it is very difficult to estimate the number of professionals involved: we estimated that around 300–400 additional professionals were working in screening centers or in collaboration with investigators.

### Variables

Likert scale questionnaires with 5 points were developed to assess: how comfortable professionals were with explaining risk categories and recommending related screening schedules; their declared understanding and expectations with risk-based breast cancer screening. Some structural socio-professional variables were also collected, namely information on professionals’ contexts of practice, demographics, country of participation to the trial, professional status within MyPeBS… Data were collected on the different steps of the trial at which respondents intervened, on their understanding of the design of the trial, on their willingness to participate, and on the information and training they received for the trial. The questionnaire would only feature questions relating to the step of the trial in which the respondents were involved. This explains how the frequency of respondents in each subsection is lower than the overall number of respondents. For each step of the trial, questions focused on professionals’ ability to communicate with women and answer their questions, on the way they deliver information, on difficulties or technical issues experienced. A greater emphasis was laid on how professionals communicate risk-estimation results and how comfortable they are with this step of the process, or how well-trained they feel to perform risk communication. Finally, data were collected on how satisfied professionals are with current population-based screening and on their views regarding a risk-based approach, and on the main issues that must be addressed before generalizing a risk-based strategy.

### Statistical analyses

Given that the participants were professionals involved in the MyPeBS study and not a random sample selected from a known target population, and that both the population and the sample were limited, inferential statistics was not considered relevant. We performed descriptive statistics on the data, without formal inferential statistical tests. Some variables were also stratified according to country or crossed with other characteristics, when relevant. Data management and analyses were performed on R 4.1.0 (R Foundation for Statistical Computing, Austria) by AR.

### Participant confidentiality and data protection

This study collected anonymous answers and did not collect information that could make participants’ identification possible. Data collected with Sphynx^®^ were stored at the University Paris 13.

## Results

### Sample description

198 professionals fully responded to the questionnaire; we can assume that the response rate of our investigation is around 20%. Eight out of ten respondents were female (Table 1), almost half of respondents were from France, and one third from Italy. The most frequent professionals are physicians (56%) with general practitioners (23%), radiologists (19%) and gynecologists (12%), the other professionals are less 7% each with nurse, radiographer, administrator, data manager, epidemiologist, medical secretary. On the whole group of respondents, 53% were working in the public sector and 10% working both in private and in public sectors.

**Table 1:** Socio-professional characteristics

Variable	% (*N*)	Number of responses
*Gender*		
Female	79.3% (157)	198
Male	20.7% (41)	
*Country of participation*		
Belgium	4% (8)	198
France	45.5% (90)	
Israel	6.6% (13)	
Italy	30.8% (61)	
Spain	7.1% (14)	
United Kingdom	6.1% (12)	
*Health-related specialty*		
Radiologist	19.2% (38)	198
Oncologist	2.5% (5)	
Gynaecologist	11.6% (23)	
General practitioner	22.7% (45)	
Biologist	1.5% (3)	
Nurse or nurse specialized in clinical research	5.1% (10)	
Radiographer (technician)	6.1% (12)	
Administrator	3% (6)	
Data / project manager	6.6% (13)	
Epidemiologist	5.1% (10)	
Medical secretary	2% (4)	
Other	14.6% (29)	
*Sector of practice*		
Private	34% (66)	198
Public	53.1% (103)	
Both	10.3% (20)	
Other	2.6% (5)	

A large portion of respondents had been previously involved in research, one fourth of them regularly and one out of ten as their main professional activity (Table 2). 60% worked in a structure dedicated to breast cancer screening and half had 30% or more of their professional activity dedicated to it. 47 out of 114 (42%) physicians performed risk estimation often in their medical practice, while 32% never or rarely did it. The sample was quite diverse in terms of start year of healthcare practice. 12% of respondents started working in the field of breast cancer screening recently, and 52.5% having started more than five years ago.

**Table 2:** Implication in research and breast cancer screening

Variable	% (*N*)	Number of Responses
*Frequency of involvement in research*		
This is the first time	22.7% (45)	198
On a few occasions	42.9% (85)	
Regularly	24.2% (48)	
Main professional role	10.1% (20)	
*Works in a structure dedicated to breast cancer screening*	59.1% (114)	198
*Proportion of breast cancer screening in professional practice*		
Less than 10%	29.6% (58)	196
10–30%	20.9% (41)	
30–50%	20.4% (40)	
More than 50%	29.1% (57)	
Frequency of performing risk estimation in professional practice*		
Never / rarely (1–2 times /year)	32.2% (36)	114
Sometimes (1–2 times /month)	16.1% (18)	
Often	42% (47)	
Not applicable / Don’t know / NR	11.4% (13)	
*Start year of professional practice*		
1976–1989	19.2% (38)	156
1990–1999	15.2% (30)	
2000–2009	24.7% (49)	
2010–2015	19.7% (39)	
*First implication in breast cancer screening*		
Less than a year ago	12% (24)	197
Between 1 year and 5 years ago	35.5% (69)	
Between 5 and 10 years ago	15% (30)	
More than 10 years ago	37.5% (74)	

*Only asked to radiologists, oncologists, gynaecologists, general practitioners and biologists

### Implication in the trial and difficulties experienced at each step

Around two-thirds of the respondents were involved in the initial steps of the trial, namely the recruitment of women, the inclusion process (checking inclusion criteria, obtaining informed consent, randomization), the administration of questionnaires, and the saliva sample collection. Breast imaging (mammography, ultrasound, or MRI) for the trial was performed by 30% of the sample, and risk communication by 58% (Table 3). Half of the respondents were in charge of the follow-up and communication with women throughout the study.

**Table 3:** Steps of intervention in mypebs (multiple answer possible)

	% Obs (*N*)
Recruitment of women: either giving initial information, contacting women, fixing appointments or leading group information meetings	66.2% (131)
Inclusion process (not including administration of questionnaires): verifying inclusion criteria, obtaining informed consent, randomisation	69.2% (137)
Administration of questionnaires: clinical data and family history questionnaire, psycho-social questionnaire	63.1% (125)
Saliva sample collection and/or logistics	65.7% (130)
Mammography, ultrasound and/or MRI	30.3% (60)
Risk consultation or communication	57.6% (114)
Follow-up and communication with women throughout the study	50% (99)
No patient-facing role / other	8.1% (16)
Total	100% (198)

Professionals generally felt quite or very comfortable answering women’s questions at each step of the trial (Table 4). For most questions, less than 3% said they were not very comfortable or not comfortable at all, it rises to 7% regarding risk communication. A large majority of professionals felt that they understood the design of the trial and the concept of breast cancer risk estimation quite or very well (respectively 93% and 95%) [see Additional file 1]. They were, however, less comfortable with the concept of genotyping based on 313 SNPs, with 38% ranking their understanding 3 or less on the 5-points scale.

**Table 4:** Difficulties with answering women’s questions at each step of the trial

Comfortable with answering women’s questions at…	Recruitment	Inclusion	Questionnaires	Saliva sample collection	Mammography	Risk communication
Women don’t ask questions at this stage	0.8% (1)	0.8% (1)	0.8% (1)	3.1% (4)	13.3% (8)	1.8% (2)
Not comfortable at all	0% (0)	0% (0)	0.8% (1)	0% (0)	1.7% (1)	0% (0)
Not very comfortable	1.5% (2)	0.8% (1)	2.4% (3)	1.6% (2)	1.7% (1)	7.1% (8)
Quite comfortable	54.2% (71)	53.5% (75)	55.2% (69)	53.1% (68)	56.7% (34)	61.1% (69)
Very comfortable	43.5% (57)	45% (60)	40.8% (51)	42.2% (54)	26.7% (16)	30.1% (34)
Total	100% (131)	100% (137)	100% (125)	100% (128)	100% (60)	100% (113)

For recruitment strategies, attitudes differed according to the country (Table 5): some professionals gave information about the trial to all women eligible for age-based screening or to all women coming for their regular mammography in the center (all the Spanish HCPs, a great majority of the British or Israeli HCPs and more than half of the Italian HCPs), while others did not offer the trial to all the women they met, thus making a selection (51% of French professionals).

**Table 5:** Selection effect when giving information about the trial

Do you give initial information about MyPeBS… % Obs (*n*)	Country						
	BEL	FRA	ISR	ITA	SPA	UK	Total
To all women eligible for age	40%(2)	33.3% (25)	80% (8)	55.2% (16)	100% (5)	71.4% (5)	46.6% (61)
To some of them that you selected	0 (0)	50.7% (38)	10% (1)	27.6% (8)	0% (0)	28.6% (2)	37.4% (49)
Only to women that come to you specifically for it or are recommended by another practitioner	60% (3)	16% (12)	10% (1)	17.2% (5)	0% (0)	0% (0)	16% (21)
Total	100% (5)	100% (75)	100% (10)	100% (29)	100% (5)	100% (7)	100% (131)

### Risk estimation: practices and needs

One of the main concerns about risk-based screening is whether professionals present the risk category in a way that women understand and that encourages discussion, and whether they themselves feel comfortable enough to do so. The means of giving back risk estimation greatly varied in this study, according to women’s risk categories (Table 6); multiple answers were allowed): for women in the high- and very high-risk categories, a majority of professionals gave back risk estimations during a face-to-face consultation (76%), but some also used remote means (36% on the phone, 16% during a teleconsultation, 9% by letter or by e-mail). For women in the moderate and low-risk categories, the respondents preferred to use the phone (respectively 55% and 52%), and face-to-face consultations (respectively 50% and 43%).

**Table 6:** Means of giving back risk Estimation according to women’s risk categories (multiple answers possible)

	for women in the high-risk and very high-risk categories	for women in the moderate-risk category	for women in the low-risk category	Total
by letter or by e-mail	8.8% (10)	18.5% (21)	26.5% (30)	26.5% (30)
by phone	36.2% (41)	54.9% (62)	52.2% (59)	63.7% (72)
during a teleconsultation	15.9% (18)	14.2% (16)	11.5% (13)	21.2% (24)
during a face-to-face consultation	76.1% (86)	50.4% (57)	42.5% (48)	76.1% (86)
other	0% (0)	0.9% (1)	0% (0)	0.9% (1)
				100% (113)

This means that the way in which risk is communicated varies from one professional to another, and from one country to another according to (i) Ethics Committees’ recommendations (ii) COVID pandemic organizational adjustments [see Additional file 2] (iii) local organizational depending on staff availability. Professionals mostly felt quite or very comfortable informing women of their risk category (89%, Table 7). However, they were less comfortable explaining high risk categories and consequent intensified screening (71%) compared to low-risk categories and the 4-years mammography interval (65%)– with 9% not feeling very comfortable or not comfortable at all explaining reduced screening. Training to deliver risk assessments was considered not sufficient or a little bit insufficient in 22% of professionals (Table 7) with 61% feeling they should sometimes or often refresh their training for the trial.

**Table 7:** Difficulties with announcing risk, explaining high and low risk categories and opinions about training and training material to give back risk estimation

	**1- Not comfortable at all**	**2**	**3**	**4**	**5-Very comfortable**	**Total**
*Informing women of their risk category*	0% (0)	0.9% (1)	10.6% (12)	52.2% (59)	36.3% (41)	100% (113)
*Explaining high risk categories*	0.9% (1)	6.2% (7)	22.1% (25)	52.2% (59)	18.6% (21)	100% (113)
*Explaining low risk category and 4-years mammography intervals*	4.5% (5)	4.5% (5)	25.9% (29)	39.3% (44)	25.9% (29)	100% (112)
	**Did not receive any training**	**Not sufficient at all**	**A little bit insufficient**	**Suffi-cient**	**Very sufficient**	**Total**
*Opinion on your training to give back risk*	*4.4% (5)*	3.5% (4)	18.6% (21)	49.6% (56)	23.9% (27)	100% (113)
	Never	Rarely	Sometimes	Often	**Total**	
*Frequency of need to refresh training for the trial*	13.1% (26)	26.3% (52)	50% (99)	10.6% (21)	100% (198)	

### Opinions on current screening and risk-based approaches

Some respondents were not very or not satisfied at all (11%) about the organization of current breast cancer screening in their respective countries (Table 8). In France, professionals tended to be more dissatisfied with the current breast cancer screening, whereas in Israel, Italy or Spain, most professionals were quite or very satisfied. This also differs according to profession: general practitioners and gynecologists tended to be the least satisfied, whereas other professions were more satisfied than the average. However, 93.5% agreed that risk-based strategies offer hope to improve breast cancer screening [see Additional file 3], with two-third strongly agreeing with this statement and with no clear differences between countries.

When asked to rank the three main concerns to be addressed before generalizing risk-based breast cancer screening [see Additional file 4], professionals most frequently chose the following items: the need to confirm the validity of statistical models to predict risk (58%), the development of a simple screening strategy (45%), the design of a public information campaign to present risk-stratified strategy (44%) and the training of healthcare professionals in risk counselling (38%).

**Table 8:** Satisfaction with current organized breast cancer screening, by country and type of professional (row percentages)

	1-Not satisfied at all	2	3	4	5-Very satisfied	Total
**Total**	1% (2)	10.1% (20)	30.3% (60)	38.9% (77)	19.7% (39)	100% (198)
**Country**						
BEL	0% (0)	12.5% (1)	37.5% (3)	37.5% (3)	12.5% (1)	100% (8)
FRA	2.2% (2)	17.8% (16)	31.1% (28)	40% (36)	8.9% (8)	100% (90)
ISR	0% (0)	0% (0)	30.8% (4)	30.8% (4)	38.5% (5)	100% (13)
ITA	0% (0)	3.3% (2)	18% (11)	41% (25)	37.7% (23)	100% (61)
SPA	0% (0)	0% (0)	50% (7)	42.9% (6)	7.1% (1)	100% (14)
UK	0% (0)	8.3% (1)	58.3% (7)	25% (3)	8.3% (1)	100% (12)
**Main professional role in MyPeBS**						
Radiologist	0% (0)	5.3% (2)	31.6% (12)	44.7% (17)	18.4% (7)	100% (38)
Oncologist	0% (0)	20% (1)	0% (0)	80% (4)	0% (0)	100% (5)
Gynaecologist	0% (0)	8.7% (2)	39.1% (9)	39.1% (9)	13% (3)	100% (23)
General practitioner	4.4% (2)	24.4% (11)	33.3% (15)	28.9% (13)	8.9% (4)	100% (45)
Biologist	0% (0)	33.3% (1)	33.3% (1)	33.3% (1)	0% (0)	100% (3)
Nurse/nurse specialised in clinical research	0% (0)	0% (0)	50% (5)	30% (3)	20% (2)	100% (10)
Radiographer (technician)	0% (0)	0% (0)	8.3% (1)	41.7% (5)	50% (6)	100% (12)
Administrator	0% (0)	0% (0)	33.3% (2)	16.7% (1)	50% (3)	100% (6)
Data/project manager	0% (0)	0% (0)	23.1% (3)	46.2% (6)	30.8% (4)	100% (13)
Epidemiologist	0% (0)	0% (0)	20% (2)	60% (6)	20% (2)	100% (10)
Medical secretary	0% (0)	0% (0)	25% (1)	0% (0)	75% (3)	100% (4)
Other	0% (0)	10.3% (3)	31% (9)	41.4% (12)	17.2% (5)	100% (29)

## Discussion

The strengths of the present study include the examination of acceptability in six programs with different organization of breast cancer screening services. The methodology was chosen to suit the research question. Professionals were involved in the construction of the questionnaire using Delphi methodology to identify the key questions, their ease and difficulty in undertaking this risk assessment and communication with women, their expectations in terms of scientific and clinical validation of this proposal, and their needs for future implementation.

The results of this study complement the findings of previous work, with the advantage that the participants are talking about a concrete experience of risk-stratified screening as part of a larger clinical trial, when previous research involved professionals talking about said risk-based screening as a hypothetical scenario. Most of the existing data was collected through in-depth methods, except three recent works [18, 20, 29], at a local or national level (Canada, France, Spain, UK, USA). No previous study exists at a multi-national level, and little is known about the variation of professionals’ perceptions on risk-based screening from one country to another. Some previous work suggests variation in perception of population-based screening and risk-based approaches according to medical specialty [4, 21]. This exploratory study shows that the need for training is identified by professionals as a critical point to consider, especially in countries where primary healthcare providers are involved in the screening program and are likely to be involved in risk-estimation communication. In addition, some national differences were observed in the opinions about current breast screening programs. Concerns regarding the implementation of risk-stratified screening highlight the need for the development of training programs and such a training could prepare healthcare professionals for any potential rollout [15]. Other studies also called for dedicated medical training on risk counselling, particularly for explaining low-risk and less frequent screening [4, 21, 30]. Specifically, professionals need to have a sufficient understanding of the concept of risk stratification and to be trained to use risk tools and communicate risk scores [24]. Relatedly, such evaluations could highlight where service redesign or reorganization is needed to facilitate risk-stratified screening, where training alone may not be sufficient. Professionals expressed the need for more specialized staff (e.g. nurses) so that they are able to answer women’s questions on risk-based screening [2, 3, 13, 15, 18, 20, 21, 29, 30].

In line with previous evidence [21, 25, 29, 30], professionals felt less at ease with increasing the interval between mammograms to four years when women were in the low-risk category, though there is evidence that 3-yearly screening in the UK is effective in the low-risk group [9]. Professionals involved in this trial were mostly confident about risk-based approaches and their potential to improve breast cancer screening, most of them called for a prospective validation of the clinical validity and utility of the models used to predict risk before generalizing this strategy to the whole population, which is indeed the purpose of the MyPeBS trial.

They also expressed concerns about the lack of simplicity of the risk-based screening strategy as compared to the current age-based strategies. They were, however, a little less concerned with some issues previously reported in the literature, such as the management and storage of personal and genetic data [4, 20], the time constraint of integrating risk counselling in medical consultations [2, 3, 13, 15, 20], the psychological impact of risk communication on women [2, 3, 13, 15, 20], or the impact on equal access to care raised by risk-based screening [3, 13, 15, 20]. These differences could be due to the setting of the present study that, for the first time, surveyed professionals involved in an ongoing pragmatic trial where risk-based screening was implemented.

## Limitations

An important limitation is the absence of direct contact with the non-investigators, and the absence of a clear information on how many professionals were reached. Moreover, those professionals that are not investigators and did not respond to the questionnaire might be less interested by risk-based approachesMoreover, French general practitioners are overrepresented as compared to the other professionals and other countries, which may bias our results. Indeed, the trial organization and the types and numbers of professionals involved differ according to countries. In some countries (Israel, Italy, UK, Spain) recruitment was performed in large screening centers, hence a reduced number of professionals were involved and expected to answer. In other cases (France, Belgium) the situation was mixed with participants recruited in numerous smaller structures, a larger number of professionals involved and more heterogeneity in their characteristics (more professionals in private practice, such as general practitioners or gynecologists, and a lesser proportion of radiologists and radiographers). Lastly, our paper only investigates opinions and practices from professionals involved in the trial, which are not necessarily representative of professionals that would be involved in the screening programs, if the risk-based strategy were to be implemented.

## Conclusions

This exploratory study is the first to assess the perspectives of professionals involved in risk-based cancer screening. It shows that a diversity of professionals is taking part in the MyPeBS trial across the 6 countries, with different implications at each step of the trial. Professionals generally felt comfortable answering women’s questions at each step of the trial. A selection bias in the recruitment of women was observed in France, but not in other countries. Professionals mostly felt comfortable informing women of their risk category but reported less confidence when explaining higher or lower risk categories. Risk communication varied from one professional to another, and from one country to another. Training to deliver risk assessments was considered insufficient by one in five respondents.

Some respondents reported dissatisfaction with current breast cancer screening, with most dissatisfaction expressed in France and among general practitioners and gynecologists. The four main reported concerns about implementation of risk-based strategies were: the need to confirm the validity of statistical models to predict risk, the development of a simple screening strategy, the design of a public information campaign to present risk-stratified strategy and the training of healthcare professionals in risk counselling. The findings indicate that to be successful, the implementation of a risk-based strategy must consider the organization of the screening system at a national level, and local differences and opinions of specific professional specialties involved in organised cancer screening. Addressing issues such as training, workforce planning and service design is essential for effective adoption.

## Electronic supplementary material

Below is the link to the electronic supplementary material.

Supplementary Material 1: Additional file 1. Methodology step-by-step diagram

Supplementary Material 2: Additional file 2. Questionnaire in English.

Supplementary Material 3: Additional file 3. Results table: Scales of understanding the main concepts of the trial. Additional file 4. Results table: Impact of Covid-19 restrictions on recruitment (multiple answers possible). Additional file 5. Results table: Agreement with the statement that risk-based approaches carry the hope of improving breast cancer screening, by country and type of professional (row percentages). Additional file 6. Results table: Main issues to address before generalizing risk-based screening.

## Data Availability

The datasets used and/or analysed during the current study are available from the corresponding author on reasonable request. The final dataset is completely anonymous. These data may be provided to reputable researchers running other research studies. The future research would be compatible with this research project and will concern professionals’ perceptions of risk-stratified screening. These organisations may be universities, local authorities or NHS organisations involved in health and care research in this country or abroad. All participant documentation reflects the future use of these data in research.

## Footnotes

1 *MyPeBS - International Randomized Study Comparing personalized*,* Risk-Stratified to Standard Breast Cancer Screening In Women Aged 40–70 -*https://www.mypebs.eu/the-project/ - coordinated by UNICANCER as sponsor.

2 The original method is based on many rounds of evaluation until a total consensus on the final questions is reached, but some adapted versions of the Delphi procedure seek to consolidate the questions and provide a final validation after two or three rounds.

3 Be an investigator - https://www.mypebs.eu/be-an-investigator/ - retrieved 2023/07/10.

## References

[1] Bank L, Jippes M, van Luijk S, den Rooyen C, Scherpbier A, Scheele F. Specialty training’s organizational readiness for curriculum change (STORC): development of a questionnaire in a Delphi study. BMC Med Educ. 2015;15:127. 26242219 10.1186/s12909-015-0408-0 PMC4525745

[2] Bellhouse S, Hawkes RE, Howell SJ, Gorman L, French DP. Breast Cancer risk assessment and primary prevention advice in primary care: A systematic review of provider attitudes and routine behaviours. Cancers. 2021;13:4150. 34439302 10.3390/cancers13164150 PMC8394615

[3] Blouin-Bougie J, Amara N, Simard J. Toward a Population-Based breast Cancer risk stratification approach? The needs and concerns of healthcare providers. JPM. 2021;11:540. 34200634 10.3390/jpm11060540 PMC8228184

[4] Bloy G, Adhéra N, Rigal L. 2015.– 7– Quand des médecins libéraux participent à une politique publique sans toujours s’y impliquer: les généralistes et le dépistage organisé des cancers. In: Meidani A, Legrand É, Jacques B, editors. La santé: du public à l’intime. Presses de l’EHESP. p 123–39.

[5] Brady SR. Utilizing and adapting the Delphi method for use in qualitative research. Int J Qualitative Methods. 2015;14:1609406915621381.

[6] Burton H, Chowdhury S, Dent T, Hall A, Pashayan N, Pharoah P. Public health implications from COGS and potential for risk stratification and screening. Nat Genet. 2013;45:349–51. 23535723 10.1038/ng.2582

[7] Dennison RA, Usher-Smith JA, John SD. The ethics of risk-stratified cancer screening. Eur J Cancer. 2023;187:1–6. 37094523 10.1016/j.ejca.2023.03.023

[8] Esserman L, Eklund M, Veer Lvt, Shieh Y, Tice J, Ziv E, et al. The WISDOM study: a new approach to screening can and should be tested. Breast Cancer Res Treat. 2021;189(3):593–8. 10.1007/s10549-021-06346-w. 34529196 10.1007/s10549-021-06346-w

[9] Evans DGR, Harkness EF, Brentnall AR, van Veen EM, Astley SM, Byers H, Sampson S, Southworth J, Stavrinos P, Howell SJ, Maxwell AJ, Howell A, Newman WG, Cuzick J. Breast cancer pathology and stage are better predicted by risk stratification models that include mammographic density and common genetic variants. Breast Cancer Res Treat. 2019;176(1):141–8. Epub 2019 Apr 2. PMID: 30941651; PMCID: PMC6548748. 30941651 10.1007/s10549-019-05210-2 PMC6548748

[10] Fernández-Domínguez JC, Sesé-Abad A, Morales-Asencio JM, Sastre-Fullana P, Pol-Castañeda S, Pedro-Gómez JE de. Content validity of a health science evidence-based practice questionnaire (HS-EBP) with a web-based modified Delphi approach. Int J Qual Health Care. 2016;28:764–73. 10.1093/intqhc/mzw106 27655793

[11] Fletcher AJ, Marchildon GP. Using the Delphi method for qualitative, participatory action research in health leadership. Int J Qualitative Methods. 2014;13:1–18.

[12] French DP, Howell A, Evans DG. Psychosocial issues of a population approach to high genetic risk identification: behavioural, emotional and informed choice issues. Breast. 2018;37:148–53. 29161653 10.1016/j.breast.2017.11.008

[13] French DP, Woof VG, Ruane H, Evans DG, Ulph F, Donnelly LS. The feasibility of implementing risk stratification into a National breast cancer screening programme: a focus group study investigating the perspectives of healthcare personnel responsible for delivery. BMC Womens Health. 2022;22:142. 35501791 10.1186/s12905-022-01730-0 PMC9063090

[14] Hasson F, Keeney S, McKenna H. Research guidelines for the Delphi survey technique. J Adv Nurs. 2000;32:1008–15. 11095242

[15] Hawkins R, McWilliams L, Ulph F, Evans DG, French DP. Healthcare professionals’ views following implementation of risk stratification into a National breast cancer screening programme. BMC Cancer. 2022;22:1058. 36224549 10.1186/s12885-022-10134-0 PMC9555254

[16] Jeon Y-H, Conway J, Chenoweth L, Weise J, Thomas TH, Williams A. 2014. Validation of a clinical leadership qualities framework for managers in aged care: a Delphi study. J Clin Nurs 12. 10.1111/jocn.12682 25209625

[17] Kerlikowske K, Bibbins-Domingo K. Toward Risk-Based breast Cancer screening. Ann Intern Med. 2021;174:710–1. 33556272 10.7326/M21-0398 PMC10365906

[18] Lapointe J, Buron A-C, Mbuya-Bienge C, Dorval M, Pashayan N, Brooks JD, Walker MJ, Chiquette J, Eloy L, Blackmore K, Turgeon A, Lambert-Côté L et al. 2022. Genet Med. 2022;24(11):2380–2388. 10.1016/j.gim.2022.08.001 10.1016/j.gim.2022.08.001 36057905

[19] Lauby-Secretan B, Scoccianti C, Loomis D, Benbrahim-Tallaa L, Bouvard V, Bianchini F, Straif K, International Agency for Research on Cancer Handbook Working Group. Breast-cancer screening– viewpoint of the IARC working group. N Engl J Med. 2015;372:2353–8. 26039523 10.1056/NEJMsr1504363

[20] Laza-Vásquez C, Hernández-Leal MJ, Carles-Lavila M, Pérez-Lacasta MJ, Cruz-Esteve I, Rué M, on behalf of the DECIDO Group. Barriers and facilitators to the implementation of a personalized breast Cancer screening program: views of Spanish health professionals. IJERPH. 2022;19:1406. 35162427 10.3390/ijerph19031406 PMC8835407

[21] McWilliams L, Woof VG, Donnelly LS, Howell A, Evans DG, French DP. Risk stratified breast cancer screening: UK healthcare policy decision-making stakeholders’ views on a low-risk breast screening pathway. BMC Cancer. 2020;20:680. 32698780 10.1186/s12885-020-07158-9 PMC7374862

[22] Moutel G, Duchange N, Darquy S, Montgolfier S, de, Papin-Lefebvre F, Jullian O, Viguier J, Sancho-Garnier H, GRED French National Cancer Institute. Women’s participation in breast cancer screening in France–an ethical approach. BMC Med Ethics. 2014;15:64. 25127662 10.1186/1472-6939-15-64 PMC4151080

[23] Parker LS, Sankar PL, Boyer J, Jean McEwen JD, Kaufman D. Normative and conceptual ELSI research: what it is, and why it’s important. Genet Med. 2019;21:505–9. 29970926 10.1038/s41436-018-0065-x

[24] Pashayan N, Antoniou AC, Ivanus U, Esserman LJ, Easton DF, French D, Sroczynski G, Hall P, Cuzick J, Evans DG, Simard J, Garcia-Closas M, et al. Personalized early detection and prevention of breast cancer: ENVISION consensus statement. Nat Rev Clin Oncol. 2020;17:687–705. 32555420 10.1038/s41571-020-0388-9 PMC7567644

[25] Rainey L, van der Waal D, Jervaeus A, Wengström Y, Evans DG, Donnelly LS, Broeders MJM. Are we ready for the challenge of implementing risk-based breast cancer screening and primary prevention? Breast. 2018;39:24–32. 29529454 10.1016/j.breast.2018.02.029

[26] van Ravesteyn NT, Schechter CB, Hampton JM, Alagoz O, Broek JJ, van den, Kerlikowske K, Mandelblatt JS, Miglioretti DL, Sprague BL, Stout NK, de Koning HJ, Trentham-Dietz A, et al. Trade-Offs between harms and benefits of different breast Cancer screening intervals among Low-Risk women. J Natl Cancer Inst. 2021;113:1017–26. 33515225 10.1093/jnci/djaa218 PMC8502479

[27] Roux A, Cholerton R, Sicsic J, Moumjid N, French DP, Giorgi Rossi P, Balleyguier C, Guindy M, Gilbert FJ, Burrion J-B, Castells X, Ritchie D, et al. Study protocol comparing the ethical, psychological and socio-economic impact of personalised breast cancer screening to that of standard screening in the my personal breast screening (MyPeBS) randomised clinical trial. BMC Cancer. 2022;22:507. 35524202 10.1186/s12885-022-09484-6 PMC9073478

[28] Sung H, Ferlay J, Siegel RL, Laversanne M, Soerjomataram I, Jemal A, Bray F. Global Cancer Statistics 2020: GLOBOCAN Estimates of Incidence and Mortality Worldwide for 36 Cancers in 185 Countries. CA: A Cancer Journal for Clinicians. 2021;71:209–249. 10.3322/caac.21660 33538338

[29] Vassy JL, Kerman BJ, Harris EJ, Lemke AA, Clayman ML, Antwi AA, MacIsaac K, Yi T, Brunette CA. Perceived benefits and barriers to implementing precision preventive care: results of a National physician survey. Eur J Hum Genet 2023:1–8. 10.1038/s41431-023-01318-8 PMC10620193 36807341

[30] Woof VG, McWilliams L, Donnelly LS, Howell A, Evans DG, Maxwell AJ, French DP. Introducing a low-risk breast screening pathway into the NHS Breast Screening Programme: Views from healthcare professionals who are delivering risk-stratified screening. Womens Health (Lond Engl). 2021 Jan-Dec;17. 10.1177/17455065211009746 10.1177/17455065211009746 PMC8060757 33877937

