# Correction: Acceptability of risk-based breast cancer screening among professionals and healthcare providers from 6 countries contributing to the MyPeBS study

**DOI:** 10.1186/s12885-025-13994-4

**Published:** 2025-04-14

**Authors:** Alexandra Roux, Lucile Hervouet, Francesca Di Stefano, David P. French, Livia Giordano, David Ritchie, Marie-Eve Rougé Bugat, Debbie Keatley, Rachel Cholerton, Lorna McWilliams, Paolo Giorgi Rossi, Corinne Balleyguier, Michal Guindy, Fiona J. Gilbert, Jean-Benoit Burrion, Marta Roman, Cécile Vissac-Sabatier, Daniel Couch, Suzette Delaloge, Sandrine de Montgolfier, MyPeBS Investigators and the MyPeBS Consortium

**Affiliations:** 1 Aix Marseille Univ, Inserm, IRD, ISSPAM, SESSTIM, Marseille, France; 2 IRIS Institut de Recherche Interdisciplinaire Sur Les Enjeux Sociaux (UMR 8156 CNRS - 997 INSERM - EHESS - UPSN), Campus Condorcet, Aubervilliers, France; 3 Piedmont Center for Cancer Epidemiology and Prevention, CPO, AOU Città Della Salute E Della Scienza Di Torino, Turin, Italy; 4 Manchester Centre for Health Psychology, University of Manchester, Manchester, UK; 5 European Cancer Leagues, Brussels, Belgium; 6 Département Universitaire de Médecine Générale, Université Toulouse 3 Paul Sabatier, Toulouse, France; 7 Independent Cancer Patient’s Voice, London, UK; 8 AUSL - IRCCS Di Reggio Emilia, Reggio Emilia, Italy; 9 Institut Gustave Roussy, Villejuif, France; 10 Assuta Medical Centers, Tel Aviv, Israel and Ben Gurion University, Beersheba, Israel; 11 University of Cambridge, Cambridge, UK; 12 Institut Jules Bordet, Brussels, Belgium; 13 IMIM (Hospital del Mar Research Institute), Barcelona, Spain; 14 Unicancer, Paris, France; 15 University of Paris Est Créteil, Créteil, France

**Correction: BMC Cancer 25, 483 (2025)**

**https://doi.org/10.1186/s12885-025–13848-z**

Following publication of the original article [1], the authors identified three typesetting errors.

- In the methodology section, second paragraph, the referenced author names “Fletcher and Marchildon” were mistakenly included. This has now been removed and the citations correctly.

- In the discussion section, paragraph two has erroneously been duplicated. This duplication has been removed.

- The in-text citations and references were misaligned. This has been corrected.

The publishers apologize for these errors. The original article [1] has been corrected.
