# Supplementary material for: Acceptability of risk-based breast cancer screening among professionals and healthcare providers from 6 countries contributing to the MyPeBS study
Source: BMC Cancer. 2025 Mar 15;25:483. doi: 10.1186/s12885-025-13848-z (PMC11910845; doi:10.1186/s12885-025-13848-z)
Supplement: Supplementary file 1 — Supplementary Material 1: Additional file 1. Methodology step-by-step diagram [file 12885_2025_13848_MOESM1_ESM.docx]

**Additional file 2. Questionnaire in English**

**Instructions:**

The whole questionnaire takes around 15-25 mn to fill in. It is preferable not to be interrupted while filling it in.

***

**Professionals involved in MyPeBS - questionnaire**

**WP5&6, USPN, IRIS**

**This project has received funding from the European Union’s Horizon 2020 research and innovation programme under grant agreement N° 755394**

*******

**Warning!** This page will automatically disconnect after a 20mn inactivity period. Please make sure to validate your answers by filling in the whole page and going to the next.

***

Thank you for taking the time to answer our questions!

Your feedback will be extremely valuable for MyPeBS itself, but also for having a better understanding of your views on risk-based screening, and its further developments.

**You can also circulate the link to the questionnaire to all the professionals from your team who are not investigators but still take part in MyPeBS (nurses, radiographers, medical secretaries, administrative staff)**

Please send any questions you might have regarding this questionnaire to: [aroux.edu@gmail.com](mailto:aroux.edu@gmail.com) and [sandrine.demontgolfier@u-pec.fr](mailto:sandrine.demontgolfier@u-pec.fr)

1. Socio-professional characteristics

1.1. What is your gender: Woman, Man, Other

1.2. In which country are you participating in MyPeBS?

- Belgium

- France

- Israel

- Italy

- Spain

- The United Kingdom

1.3. What is your main professional status within MyPeBS?

- - - - radiologist
      - oncologist,
      - gynaecologist,
      - general practitioner,
      - biologist,
      - nurse or nurse specialised in clinical research,
      - radiographer (technician),
      - administrator,
      - data / project manager,
      - epidemiologist,
      - medical secretary
      - other (Please specify)

1.4. When did you start your professional practice in the medical field? (scrolling menu)

1.5. What is your sector of practice?

- - - Private
    - Public
    - Both
    - Other (please specify):

1.6. In your professional life, how often did you participate in research programs or in clinical trials?

- - - Never (this is the first time)
    - On a few occasions
    - Regularly
    - This is my main professional role
    - Other (Please specify):

1.7. (Filter: only HCP) How often do you perform (any kind of) risk-estimation in your medical practice?

- - Never
  - Rarely (1-2 per year)
  - Sometimes (1-2 times per month)
  - Often
  - Not applicable
  - I don’t know

1.8. Do you work in a structure dedicated to population-based breast cancer screening? - Yes

- No
- I don’t know

1.9. What is the proportion of your current professional activity that you dedicate to breast cancer screening?

- - Less than 10 %
  - Between 10-30 %
  - Between 30-50 %
  - More than 50 %.

1.10. When did you first become involved in breast cancer screening?

- - Less than a year ago
  - Between 1 year and 5 years ago
  - Between 5 and 10 years ago
  - More than 10 years ago

1.11. What are the most frequent resources you use for your medical education or training on breast cancer screening? (Please rank the three main resources)

- - - - Medical journals
      - Scientific and institutional guidelines
      - Pharmaceutical industry training sessions
      - Training sessions in the public / private sector
      - Medical or scientific conferences
      - Medical sales representatives
      - Not concerned
      - Other (please specify)

2. MyPeBS : overall view

2.1. In which step(s) of MyPeBS are you involved? (Please tick all that apply):

- Recruitment of women: either giving initial information, contacting women, fixing appointment or leading group information meeting
- Inclusion process (except administration of questionnaire): inclusion criteria, information and consent form, randomisation
- Administration of questionnaires: clinical data and family history questionnaire, psycho-social questionnaire
- Saliva sample collection and/or logistics
- Mammography, ultrasound and/or MRI
- Risk consultation or communication
- Follow-up and communication with women throughout the study
- No patient-facing role or other

NB: this is a “filter” question. The dedicated questions regarding each specific step of the trial (section 4 of the questionnaire) will only appear if the person declared to be involved in the step.

2.2. Regarding your participation to MyPeBS, would you say that you work in MyPeBS

- It is part of your routine work or you were assigned to it?
- You volunteered or decided to become an investigator in your medical structure?
- You were recruited for it?
- Other (please specify):

2.3. On a scale from 1-5, how well did you understand the design of the trial (randomisation, two arms)? (scale: Not at all → Very well)

2.4. On a scale from 1-5, how well did you understand the concept of breast cancer risk estimation? (scale: Not at all → Very well)

2.5. On a scale from 1-5, how well did you understand genotyping based on 313 SNPs / DNA polymorphisms? (scale: Not at all → Very well)

2.6. What kind of information or training did you receive for MyPeBS? (please tick all that apply)

- - Written information (leaflet, web)
  - Oral information or presentation of the study
  - MyPeBS e-learning tool or module provided by Unicancer
  - MyPeBS tutorial or online videos provided by Unicancer
  - Self-training
  - Other (please specify):

(If they followed the e-learning) About the MyPeBS e-learning tool or module provided by Unicancer

2.6.1. …on a scale from 1-5, how satisfied are you with the e-learning material: (scale Not satisfied at all → Very satisfied)

2.6.2. …how difficult did you find the evaluation quiz? (Scale Very difficult → Very easy)

2.6.3. …how did you find the length of the e-learning?

- Too short

- Too long

- Appropriate

- I don't know

2.6.4. Do you have suggestions on how to improve the training material, or was any information missing? (open question)

2.7. After your initial training, did you feel the need to refresh your information on the trial?

- - Never
  - Rarely
  - Sometimes
  - Often

3. Your journey in MyPeBS

*(NB: all the sections are filtered according to the answers to the question 2.1.: we only ask the set of questions that corresponds to the specific steps they said they were involved in)*

3.1. Recruitment of women: either giving initial information, contacting women, fixing appointment or leading group information meeting

3.1.1. Do you give initial information about MyPeBS…

- - To all women eligible for age
  - To some of them that you selected
  - Only to women that come to you specifically for it or are recommended by another practitioner
  - Not applicable

3.1.2. If you give initial information, how do you do it? (please tick all that apply)

- - - By oral communication: during the consultation or mammography appointment
    - By oral communication: explanations given in the waiting room
    - By oral communication: by phone
    - By written information: sent to women (letter, e-mail)
    - By written information: given at the consultation or mammography appointment
    - By written information: available in the waiting room

3.1.3. Among the women who receive information about the trial, how would you estimate the rate of participation in the study?

- All of them or almost all of them accept to participate (more than 90 %)
- Most of them accept to participate (60-90 %)
- Half of them accept to participate (40-60 %)
- Some of them accept to participate (10-40 %)
- Very few or none of them accept to participate (less than 10 %)
- I don’t know

3.1.4. On a scale from 1-5, how comfortable are you with explaining the trial to women? (Scale: Not comfortable at all —> very comfortable)

3.1.5. If women ask questions about the trial during this step of the process, how comfortable are you with answering their questions?

- - Women don’t ask questions at this stage of the trial
  - Not comfortable at all
  - Not very comfortable
  - Quite comfortable
  - Very comfortable

3.1.6. Did you experience difficulties to recruit women?

- Never

- Rarely

- Sometimes

- Often

- Almost all the time or all the time

3.1.6.1. If you experienced difficulties to recruit women, can you explain why? (open question)

3.2. Inclusion process (except administration of questionnaire): inclusion criteria, information and consent form, randomisation

3.2.1. What is the frequency of inclusion at your workplace?

- - Less than one woman per month
  - Between 1 and 5 women per month
  - Between 5 and 30 women per month
  - Between 30 and 50 women per month women per month
  - More than 50 women per month
  - Other (please specify)

3.2.2. How often do women react negatively when they are not in the stratified-risk arm? - Never

- Rarely

- Sometimes

- Often

- Almost all the time or all the time

3.2.3. On a scale from 1-5, how comfortable are you with the inclusion step of the trial? (Scale: Not comfortable at all —> Very comfortable)

3.2.4. If women ask questions about the trial during this step of the process, how comfortable are you with answering their questions?

- - Women don’t ask questions at this stage of the trial
  - Not comfortable at all
  - Not very comfortable
  - Quite comfortable
  - Very comfortable

3.2.5. At the inclusion step, how often did you experience problems with the online web platform (availability, bugs…)?

- Never

- Rarely

- Sometimes

- Often

- Almost all the time or all the time

3.2.6. At the inclusion step, how often did you experience problems with space management when women fill in questionnaires?

- Never

- Rarely

- Sometimes

- Often

- Almost all the time or all the time

3.3. Administration of questionnaires: clinical data and family history questionnaire, psycho-social questionnaire

3.3.1. How do women actually fill in the inclusion questionnaires? (Please tick all that apply)

- - With a tablet at your workplace
  - With a computer at your workplace
  - With their smartphone at your workplace
  - With a tablet / computer / smartphone at home
  - Other (please specify)

3.3.2. How often do you help women filling in the questionnaires?

- Never

- Rarely

- Sometimes

- Often

- Almost all the time or all the time

3.3.3. If women ask questions about the trial during this step of the process, how comfortable are you with answering their questions?

- - Women don’t ask questions at this stage of the trial
  - Not comfortable at all
  - Not very comfortable
  - Quite comfortable
  - Very comfortable

3.4. Saliva sample collection and/or logistics

3.4.1. Did you encounter any of the following problems? (please tick all that apply)

- - Not enough tubes
  - Covid-19 restrictions
  - Technical difficulties to close the tubes
  - Not enough space in the workplace to collect the sample
  - Difficulties for women to spit in the tube, or to produce enough saliva
  - Difficulties with samples organisation or storage
  - Logistic difficulties with sending the tubes
  - Other (please specify):

3.4.2. If women ask questions about the trial during this step of the process, how comfortable are you with answering their questions?

- - Women don’t ask questions at this stage of the trial
  - Not comfortable at all
  - Not very comfortable
  - Quite comfortable
  - Very comfortable

3.5. Mammography and/or ultrasound

3.5.1. If women ask questions about the trial during this step of the process, how comfortable are you with answering their questions?

- - Women don’t ask questions at this stage of the trial
  - Not comfortable at all
  - Not very comfortable
  - Quite comfortable
  - Very comfortable

3.6. Risk consultation or communication

3.6.1. Table: How do you communicate the result of this risk estimation… (please tick all that apply)

|  | ...for women in the high-risk and very high-risk categories | ...for women in the moderate-risk category | ...for women in the low-risk category |
| --- | --- | --- | --- |
| by letter or by e-mail |  |  |  |
| by phone |  |  |  |
| during a  teleconsultation |  |  |  |
| during a face-to-face consultation |  |  |  |
| other (please specify): |  |  |  |

3.6.2. On a scale from 1-5, how comfortable are you with informing women of their risk category? (Scale: Not comfortable at all —> Very comfortable)

3.6.3. If women ask questions about the trial during this step of the process, how comfortable are you with answering their questions?

- - Women don’t ask questions at this stage of the trial
  - Not comfortable at all
  - Not very comfortable
  - Quite comfortable
  - Very comfortable

3.6.4. How would you qualify your training to give back such a risk estimation?

- - I did not receive any training
  - Not sufficient at all
  - A little bit insufficient
  - Sufficient
  - Very sufficient

3.6.5. On a scale from 1-5, how easy do you find explaining to women the high risk category? (Scale: Very difficult —> Very easy)

3.6.6. On a scale from 1-5, how easy do you find explaining to women the low risk category and 4-years mammography intervals? (Scale: Very difficult —> Very easy)

3.6.7. On a scale from 1-5, how clear did you find the risk result sheet to be given to women or accessible on the web platform? (Scale: Not clear at all —> Very clear)

3.6.7.1. If it was not clear enough, how would you improve it? (Open question)

3.6.8. Did you access and watch the MyPeBS videos for risk announcement consultations?

- Yes

- No

- I don’t know

3.6.8.1. (If yes) On a scale from 1-5, how useful did you find these videos?(Scale Useless → Very Useful)

3.6.8.2. If you found them useless, could you explain why? (Open question)

3.7. Follow-up and communication with women throughout the study

3.7.1. To your knowledge, did some women you enlisted quit the trial?

3.7.2. (if yes) Did some women expressed any of the following reasons for quitting the trial? (Please tick all that apply)

- - - Disappointed about being in the standard arm instead of the risk-stratified arm
    - Lack of time or too burdensome
    - Moving elsewhere
    - Anxiety about the trial
    - Difficulty to interact with the web platform
    - Difficulty to understand the proposition of follow-up
    - I don’t know
    - Other (please specify):

4. Opinions on breast cancer screening and its further developments

4.1. On a scale from 1-5, how satisfied are you with the current population-based breast cancer screening (in your country)? (Scale: Not satisfied at all —> Very satisfied)

4.2. Do you think that the risk-based approach carries the hope of improving breast cancer screening? (Scale: Strongly disagree —> Strongly agree)

4.3. According to you, what are, in the following list, the three main concerns we have to address before generalising the risk-based approach to the whole population? (Hierarchise the 3 more relevant items)

- - - Confirmation of the validity of statistical models to predict risk
    - Training of healthcare professionals in risk counselling
    - Time management to explain risk-based screening during consultations
    - Management and storage of personal and genetic data
    - Design a public information campaign to present the risk-stratified screening strategy
    - Recruiting extra staff to deliver information and answer women's question about the screening system
    - Evaluate of the psychological issues raised by this new proposition
    - Logistics of implementing the risk-based approach into existing population-based screening
    - Develop a screening strategy simple enough so it can be efficiently explained to and followed by all women
    - Equity in the access to screening
    - Other (Please specify) :

5. General satisfaction and feedback on MyPeBS

5.1. On a scale from 1-5, how satisfied are you with the MyPeBS trial? (Scale: Not satisfied at all —> Very satisfied)

5.2. Which communication tools do you use to present MyPeBS to women?

(Please tick all that apply)

- - None
  - Online videos on the MyPeBS website
  - Flyer
  - Leaflet
  - Posters
  - Online booklet on the MyPeBS website
  - Information and consent forms
  - Other (please specify):

5.3. On a scale from 1-5, how satisfied are you with the communication tools provided by MyPeBS? (scale: Not satisfied at all → Very satisfied)

5.4. How did the Covid-19 measures impact your recruitment process or the way you imagined the recruitment process ? (please tick all that apply)

- - - Information is now given remotely (by phone, by e-mail or by letter)
    - Decrease in the frequency of recruitment
    - We had to find a new place for women to fill in questionnaires
    - Women now fill in questionnaires partially or totally at home
    - I/We stopped the recruitment temporarily
    - I/We stopped the recruitment permanently
    - Other (specify)

5. 5. Did you experience difficulties at any step of the trial or do you have any suggestions to improve the trial process, the training for the trial, the communication tools? Do you have any specific needs in terms of training, or communication material? (open question)
