# Supplementary figures and images for: Acceptability of risk-based breast cancer screening among professionals and healthcare providers from 6 countries contributing to the MyPeBS study
Source: BMC Cancer. 2025 Mar 15;25:483. doi: 10.1186/s12885-025-13848-z (PMC11910845; doi:10.1186/s12885-025-13848-z)

**Additional file
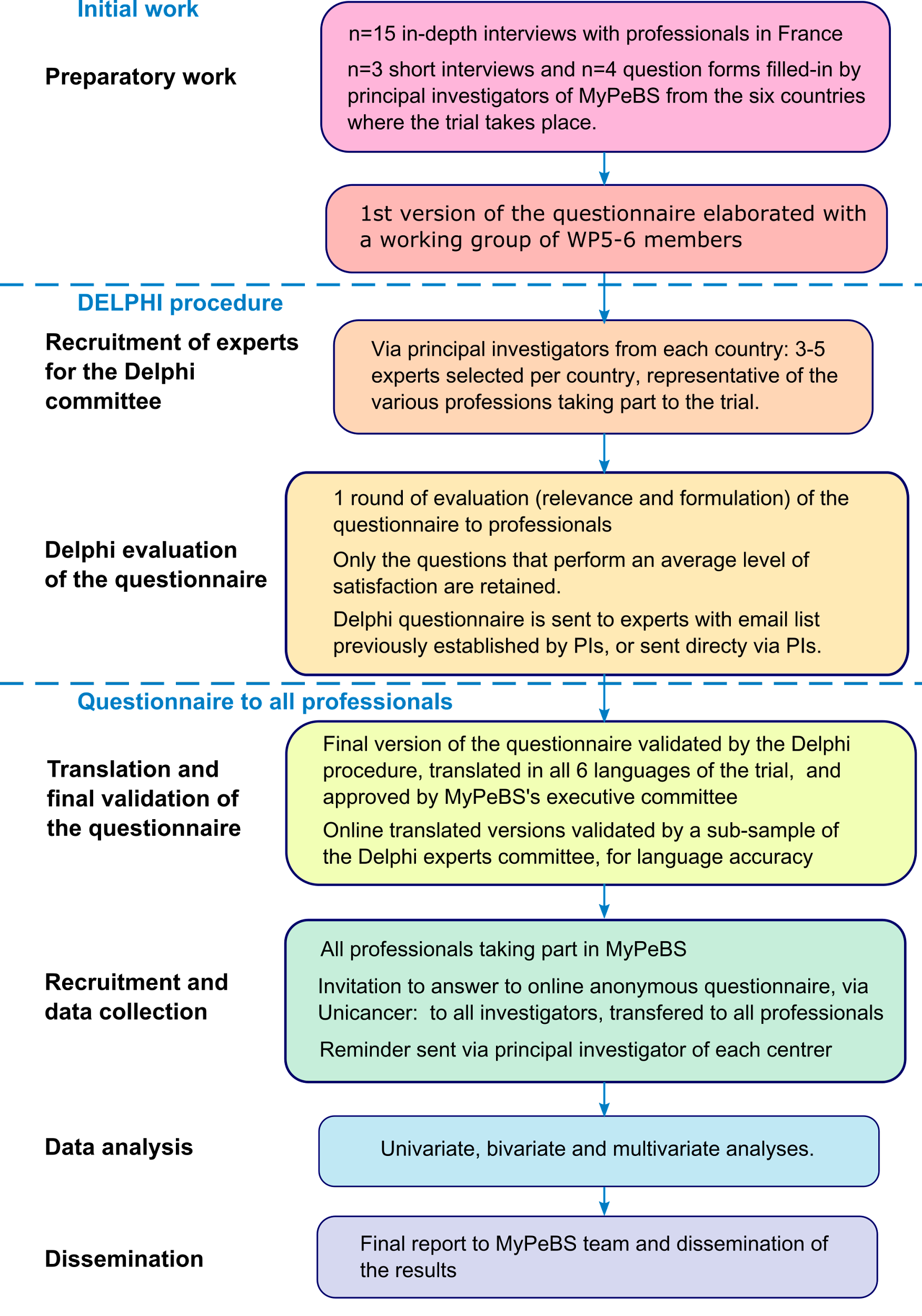
1:Methodology step-by-step diagram**

Supplement: Supplementary file 2 — Supplementary Material 2: Additional file 2. Questionnaire in English. [file 12885_2025_13848_MOESM2_ESM.docx]
