# Supplementary material for: Acceptability of risk-based breast cancer screening among professionals and healthcare providers from 6 countries contributing to the MyPeBS study
Source: BMC Cancer. 2025 Mar 15;25:483. doi: 10.1186/s12885-025-13848-z (PMC11910845; doi:10.1186/s12885-025-13848-z)
Supplement: Supplementary file 3 — Supplementary Material 3: Additional file 3. Results table: Scales of understanding the main concepts of the trial. Additional file 4. Results table: Impact of Covid-19 restrictions on recruitment (multiple answers possible). Additional file 5. Results table: Agreement with the statement that risk-based approaches carry the hope of improving breast cancer screening, by country and type of professional (row percentages). Additional file 6. Results table: Main issues to address before generalizing risk-based screening. [file 12885_2025_13848_MOESM3_ESM.docx]

**Additional file 3. Results Table: Scales of understanding the main concepts of the trial**

| How well did you understand… | **1-Not at all** | **2** | **3** | **4** | **5-Very well** | **Total** |
| --- | --- | --- | --- | --- | --- | --- |
| … the design of the trial (randomisation, two arms)? | 0.5% (1) | 2%  (4) | 4.1% (8) | 12.2% (24) | 81.1% (159) | 100% (196) |
| … the concept of breast cancer risk estimation? | 1.5% (3) | 0.5% (1) | 2.5% (5) | 24.7% (49) | 70.7% (140) | 100% (198) |
| … genotyping based on 313 SNPs/DNA polymorphisms? | 2.5%  (5) | 12.6% (25) | 23.2% (46) | 32.3% (64) | 29.3% (58) | 100% (198) |

**Additional file 4. Results Table: Impact of Covid-19 restrictions on recruitment (multiple answers possible)**

| Impact Covid-19 on recruitment: | **% Obs. (N)** |
| --- | --- |
| Information is now given remotely (by phone, by e-mail or by letter) | 20.7% (39) |
| Decrease in the frequency of recruitment | 55.9% (105) |
| We had to find a new place for women to fill in questionnaires | 5.9% (11) |
| Women now fill in questionnaires partially or totally at home | 10.6% (20) |
| I/We stopped the recruitment temporarily | 55.9% (105) |
| I/We stopped the recruitment permanently | 2.7% (5) |
| Other | 10.1% (19) |
| Total | 100% (188) |

**Additional file 5. Results Table: Agreement with the statement that risk-based approaches carry the hope of improving breast cancer screening, by country and type of professional (row percentages)**

|  | **Strongly disagree** | **Disagree** | **Neutral** | **Agree** | **Strongly agree** | **Total** |
| --- | --- | --- | --- | --- | --- | --- |
| **Total** | **0% (0)** | **0% (0)** | **6.6% (13)** | **27.3% (54)** | **66.2% (131)** | **100% (198)** |
| **Country** | | | | | | |
| BEL | 0% (0) | 0% (0) | 12.5% (1) | 12.5% (1) | 75% (6) | 100% (8) |
| FRA | 0% (0) | 0% (0) | 4.4% (4) | 21.1% (19) | 74.4% (67) | 100% (90) |
| ISR | 0% (0) | 0% (0) | 0% (0) | 23.1% (3) | 76.9% (10) | 100% (13) |
| ITA | 0% (0) | 0% (0) | 9.8% (6) | 37.7% (23) | 52.5% (32) | 100% (61) |
| SPA | 0% (0) | 0% (0) | 7.1% (1) | 28.6% (4) | 64.3% (9) | 100% (14) |
| UK | 0% (0) | 0% (0) | 8.3% (1) | 33.3% (4) | 58.3% (7) | 100% (12) |
| **Main professional role in MyPeBS** | | | | | | |
| Radiologist | 0% (0) | 0% (0) | 10.5% (4) | 21.1% (8) | 68.4% (26) | 100% (38) |
| Oncologist | 0% (0) | 0% (0) | 0% (0) | 0% (0) | 100% (5) | 100% (5) |
| Gynecologist | 0% (0) | 0% (0) | 0% (0) | 26.1% (6) | 73.9% (17) | 100% (23) |
| General practitioner | 0% (0) | 0% (0) | 8.9% (4) | 22.2% (10) | 68.9% (31) | 100% (45) |
| Biologist | 0% (0) | 0% (0) | 0% (0) | 33.3% (1) | 66.7% (2) | 100% (3) |
| Nurse or nurse specialized in clinical research | 0% (0) | 0% (0) | 0% (0) | 40% (4) | 60% (6) | 100% (10) |
| Radiographer (technician) | 0% (0) | 0% (0) | 8.3% (1) | 33.3% (4) | 58.3% (7) | 100% (12) |
| Administrator | 0% (0) | 0% (0) | 0% (0) | 16.7% (1) | 83.3% (5) | 100% (6) |
| Data / project manager | 0% (0) | 0% (0) | 7.7% (1) | 30.8% (4) | 61.5% (8) | 100% (13) |
| Epidemiologist | 0% (0) | 0% (0) | 10% (1) | 50% (5) | 40% (4) | 100% (10) |
| Medical secretary | 0% (0) | 0% (0) | 0% (0) | 75% (3) | 25% (1) | 100% (4) |
| Other | 0% (0) | 0% (0) | 6.9% (2) | 27.6% (8) | 65.5% (19) | 100% (29) |

**Additional file 6. Results Table: Main issues to address before generalizing risk-based screening**

|  | **Importance** | **% Obs (N)** |
| --- | --- | --- |
| Confirmation of the validity of statistical models to predict risk | 1.59 | 58% (102) |
| Training of healthcare professionals in risk counselling | 0.7 | 37.5% (66) |
| Time management to explain risk-based screening during consultations | 0.4 | 19.9% (35) |
| Management and storage of personal and genetic data | 0.28 | 15.3% (27) |
| Design a public information campaign to present the risk-stratified screening strategy | 0.81 | 43.8% (77) |
| Recruiting extra staff to deliver information and answer women's question about the screening system | 0.18 | 10.8% (19) |
| Evaluation of the psychological issues raised by this new proposition | 0.27 | 16.5% (29) |
| Logistics of implementing the risk-based approach into existing population-based screening | 0.42 | 25% (44) |
| Development of a screening strategy simple enough so it can be efficiently explained to and followed by all women | 0.86 | 44.9% (79) |
| Equity in the access to screening | 0.33 | 18.8% (33) |
| Other | 0.08 | 3.4% (6) |
| Total |  | 100% (176) |

Note: The question was “rank the three main issues we need to address before generalizing risk-based screening to a population level”**.** The importance is calculated as the average rank which the modality was quoted.
